# Assessment of macular choroidal and retinal thickness: a cohort study in Tibetan healthy children

**DOI:** 10.1038/s41598-024-51949-0

**Published:** 2024-01-16

**Authors:** Yao Yao, Jing Fu, Jiawen Liu, Lei Li, Weiwei Chen, Zhaojun Meng

**Affiliations:** 1 Beijing Key Laboratory of Ophthalmology & Visual Sciences, Beijing Tongren Eye Center, Beijing Tongren Hospital, Capital Medical University, Beijing, 100730 China; 2 Department of Industrial Engineering and Operation Research, University of California, Berkeley, Berkeley, USA

**Keywords:** Paediatrics, Public health, Health care, Epidemiology, Paediatric research

## Abstract

This research investigates the distribution, progressive changes, and contributing factors of macular choroidal and retinal thickness in Tibetan children utilizing swept-source optical coherence tomography (SS-OCT). The Lhasa childhood study recruited 1632 students from seven primary schools in Lhasa. These participants underwent OCT and ophthalmological evaluations, encompassing retinal and choroidal thickness measurements, refractive error, axial length (AL), and systemic examinations. The median age of the scholars was 8.57 ± 0.50 years with a median spherical equivalent (SE) of 0.19 ± 1.28D. Multivariate regression analysis revealed that thinner macular choroid thickness was correlated with lower value of SE, worse best-corrected visual acuity, higher mean arterial blood pressure (MABP) and boys, while retinal thickness was associated with better image quality and lower value of SE. The choroid and retina were significantly thinner in myopic children. SE was positively related to the thickness of all choroidal and full retinal subregions. In comparison to baseline data from 20 months prior, most regions of the full retina had significantly thinned. Choroidal thickness of Tibetan children is thinner than that of same-age children from other regions. Thinning of retina, the outer-sector GCC and GCIPL may be specified as a follow-up and prognostic indicator for myopia.

## Introduction

The choroid, a highly vascularized structure within the ocular anatomy, performs a multitude of physiological functions, including nourishing the retina and optic nerve, forming the retinal barrier, absorbing light, and regulating intraocular pressure and ocular growth. Choroidal thickness (ChT) is deemed a sensitive biomarker for the prediction, diagnosis, intervention, and monitoring of various acute or chronic retinal and choroidal diseases in children, particularly in myopia^1,2^. Choroidal changes are temporary and prompt, suggesting that ChT can serve as an early onset and potentially reliable parameter of the defocusing effect^3^. Various studies have indicated that subfoveal ChT is influenced by factors such as age, race, sex, axial length, and intraocular pressure^4,5^. Similarly, a correlation between retinal thickness and refractive error has been found that retinal thickness decreases with increased myopia degree and axial length enlongation^6,7^. This relationship is also reflected in different layers of the retina, such as the ganglion cell complex (GCC) and ganglion cell inner plexiform layer (GCIPL)^8,9^.

The advent of optical coherence tomography (OCT) has revolutionized our understanding of the morphological structure of the vitreous body and retina through non-invasive examination techniques^10^. With the rapid advancement of OCT technology, the emergence of swept-source OCT (SS-OCT) has enhanced the quality and visualization of choroidal imaging. This method’s built-in automatic segmentation system also aids us in further observing and analyzing the morphological structure and thickness changes in the retina and choroid layers^11^. Compelling evidence illustrates that SS-OCT can accurately quantify the hierarchical structure of the retina and choroid during disease progression. For instance, studies employing SS-OCT have also demonstrated a gradual decrease in choroidal thickness with increasing axial length in individuals with high myopia^12,13^. Furthermore, by utilizing OCT to evaluate the thickness of the macular retina, RNFL and peripapillary structures, we can predict the progression of glaucoma^14,15^. Thus, novel imaging technologies will further expand our understanding of ocular diseases.

Several studies have reported and compared differences in choroidal and retinal thickness between different ethnicities^16–18^. Nonetheless, the thickness of the retina and choroid in Tibetan Chinese children remains largely unexplored. Lhasa, Tibet, the loftiest region in China, is home to a vast Tibetan populace. Its intense light conditions^19,20^, reduced oxygen levels^21,22^, and unique lifestyle like a high-calorie, single-type diet^23^ and relatively light schoolwork pressure could potentially influence children’s ocular growth. Regrettably, there is presently no ocular database of retina, choroid and eye development information for Tibetan children. Consequently, this study was conceived to assess the macular choroidal and retinal thickness in Tibetan children utilizing SS-OCT. We further investigated the impact of various associated factors on retinal and choroidal thickness through the detailed systematic and ophthalmic examination. These discoveries assist us in validating the developmental patterns of the retina and choroid in children.

## Results

### General characteristics of the study

Owing to loss of follow-up, a total of 1632 children were incorporated in the OCT assessment, of whom 37 failed to complete the full examination; thus, a 90% response rate was achieved. After excluding images with low quality or significant abnormalities, 1555 children met the inclusion criteria. No significant gender or ethnic differences between the included and excluded samples were observed. The average age of the students involved in the assessment was 8.57 ± 0.50 years; 47.8% were female, and 95.1% were Tibetan. Table 1 showcases the systemic and ocular characteristics of all participating students, with an SE of 0.19 ± 1.28D. The percentages of children with myopia, emmetropia, and hyperopia were 22.3%, 27.8%, and 49.9%, respectively.

**Table 1:** Systemic and ocular parameters in Tibetan children between sexes.

Characteristics	Average	Boys	Girls	P value
Age	8.58 ± 0.47	8.59 ± 0.45	8.56 ± 0.49	0.52
Height	130.5 ± 6.00	130.22 ± 5.70	130.06 ± 6.32	< 0.001*
Weight	27.25 ± 5.51	27.79 ± 5.84	26.65 ± 5.05	< 0.001*
BMI	16.00 ± 2.45	16.30 ± 2.66	15.67 ± 2.16	0.40
Heart rate	91.42 ± 14.20	91.55 ± 14.11	91.29 ± 14.31	0.73
SBP	96.21 ± 10.32	97.27 ± 10.65	95.05 ± 9.83	< 0.001*
DBP	61.53 ± 9.69	62.17 ± 9.84	60.84 ± 9.47	0.007*
MABP	73.09 ± 8.96	73.87 ± 9.12	72.24 ± 8.71	< 0.001*
Blood oxygen saturation	90.47 ± 3.62	90.34 ± 3.47	90.61 ± 3.77	0.14
DS	0.58 ± 1.29	0.55 ± 1.27	0.61 ± 1.31	0.40
DC	− 0.75 ± 0.83	− 0.74 ± 0.83	− 0.77 ± 0.82	0.53
SE	0.20 ± 1.27	0.18 ± 1.27	0.23 ± 1.28	0.52
Axial length	22.78 ± 0.79	23.02 ± 0.76	22.52 ± 0.74	< 0.001*
Uncorrected VA	0.13 ± 0.19	0.12 ± 0.20	0.13 ± 0.19	0.24
Near VA	− 0.10 ± 0.14	− 0.11 ± 0.14	− 0.10 ± 0.14	0.51
BCVA	0.01 ± 0.08	0.01 ± 0.08	0.02 ± 0.07	0.44
Intraocular pressure	15.25 ± 3.02	15.11 ± 3.03	15.40 ± 3.00	0.06*
Stereoacuity	1(1, 2)	1(1, 2)	1(1, 2)	0.82

All data were listed as mean ± standard deviation for continuous variables and as median (quartile) for category variables.

*BMI* body mass index, *SBP* systolic blood pressure, *DBP* diastolic blood pressure, *MABP* mean arterial blood pressure, *VA* visual acuity, *BCVA* best-corrected visual acuity.

*P < 0.05, statistical differences between sexes.

### Distribution of macular full retina, GCC, GCIPL, RNFL, and choroid thickness

**Figure 1:**
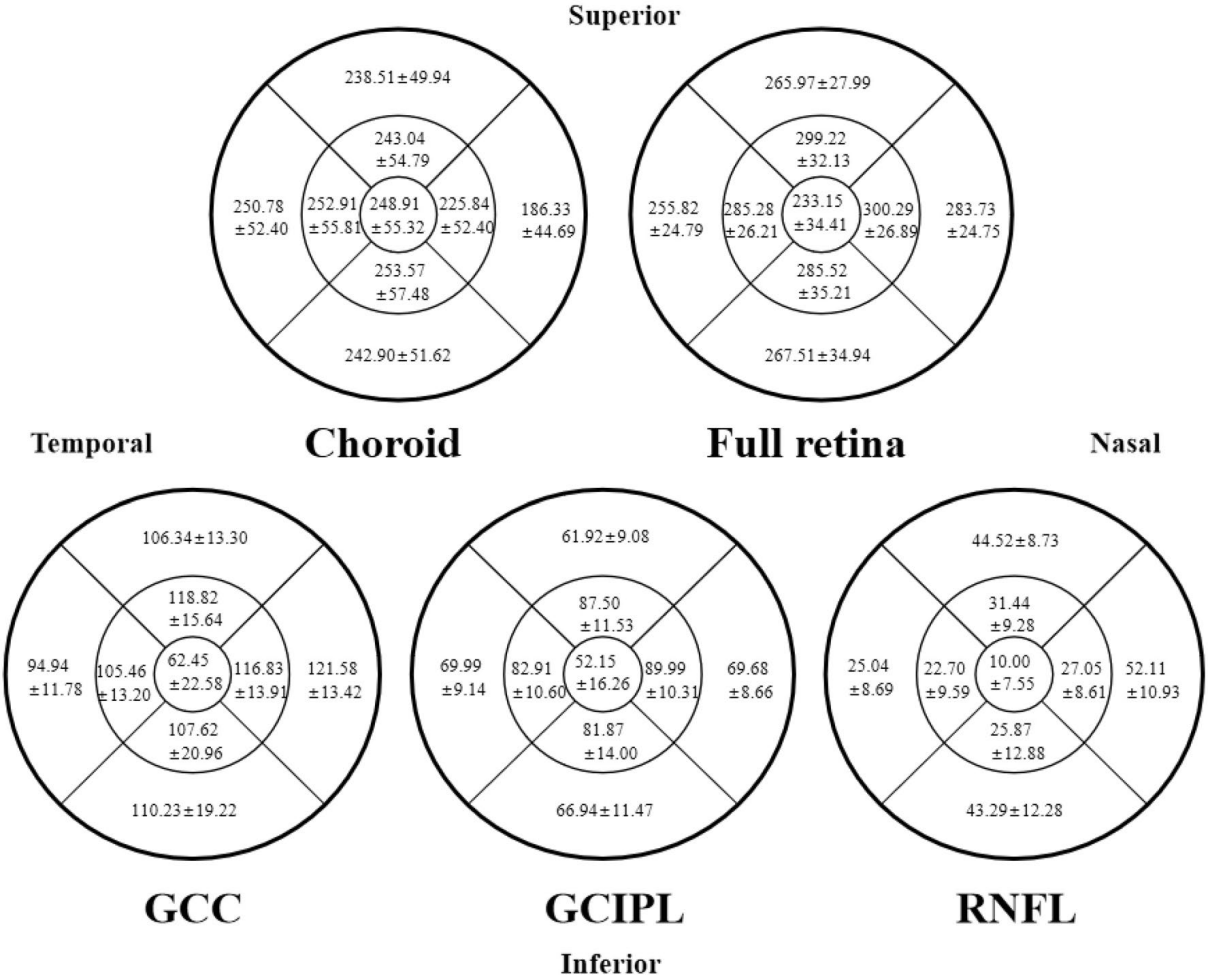
Thickness distribution maps of macular layers. Topographic distributions of macular choroid, full retina, ganglion cell-inner plexiform layer, ganglion cell complex and retinal nerve fiber layer in different sectors are shown through the picture. The mean ± SD values (lm) are presented.

Figure 1 illustrates the distribution of thickness. The mean thickness of the macular choroid, full retina, GCC, GCIPL, and RNFL was 233.23 ± 44.40 μm, 272.72 ± 24.32 μm, 107.91 ± 11.37 μm, 70.82 ± 7.02 μm, and 37.21 ± 6.64 μm, respectively. In both the inner and outer annulus of the choroid, the nasal region was the thinnest, succeeded by the superior, while the temporal and inferior regions were comparatively thicker. Conversely, the distribution of all retinal layers was significantly thinner in the temporal and inferior regions but thickest in the nasal region.

Excluding the inferior outer annulus region, the ChT of all other regions was significantly thicker in girls than in boys (P < 0.05). In terms of mean thickness, full retina and RNFL were not significantly different between genders, but girls still had marginally thicker GCC and GCIPL layers. The detailed distribution of each sub-region and the comparison between genders are depicted in Supplementary Table 1. Analysis of covariance revealed no significant difference in choroidal thickness (F = 0.06, P = 0.80) between the genders after eliminating the confounding effect of AL and IOP, but still a difference in retinal thickness (F = 9.54, P = 0.002).

In the refractive sub-groups analysis of emmetropia, hyperopia, and myopia, the ChT was significantly different between groups (P < 0.001). The ChT and retinal thickness in the myopia group were significantly lower than in the hyperopic and emmetropic groups. Regarding full retinal thickness, only the foveal and inner-inferior regions were not significantly different between refractive groups. For the thickness of GCIPL and GCC, the outer-annulus region was significantly different between groups (P < 0.05) but not the inner-annulus. There were inter-group differences in RNFL in the nasal region but not in other regions or averaged thickness, as displayed in Supplementary Table 1. Under the condition of eliminating the effects of AL and IOP, there were still significant differences in choroidal (F = 34.49, P < 0.001) and retinal thickness (F = 6.36, P = 0.002) between different refractive groups.

### Factors influencing macular thickness

Univariate linear regression was used to analyze the effects of systemic and ocular factors on the thickness of each layer of the choroid and retina, and those with P > 0.1 were included in the multivariate regression analysis. Considering the problem of covariance among variables, we included SE (excluding AL), BCVA (excluding stereoacuity), height (excluding weight) in the multivariate regression model. Multivariate linear regression analysis revealed that macular choroid thickness was associated with SE (P < 0.001, r = 0.27), BCVA (P < 0.001, r = 0.08), MABP (P = 0.008, r = 0.05) and gender (P = 0.011, r = 0.06). Conversely, macular retinal and stratified thickness were primarily correlated with image quality and SE (P < 0.01 for all). Thickness of GCIPL, GCC and RNFL layers were all correlated with BCVA, while GCC was also positively correlated with height (P < 0.05 for all) (Table 2). The correlation between image quality and retinal thickness may be due to the clarity of the image, which affects the accuracy of the segmentation the measured thickness.

**Table 2:** Multiple linear regression analysis between macular thickness and systemic parameters.

Macular layers		Unstandardized coefficient	Standard error	Standardized coefficient	P value	VIF	r value
Choroid	Gender	5.483	2.142	0.063	0.011	1.013	0.06
	SE	10.027	0.840	0.294	0.000	1.019	0.27
	Age	3.444	2.309	0.037	0.136	1.036	0.04
	BCVA	70.426	14.787	0.117	0.000	1.019	0.08
	IOP	0.661	0.354	0.046	0.062	1.014	0.05
	MABP	0.324	0.121	0.067	0.008	1.045	0.05
Retina	Image quality	1.024	0.081	0.302	0.000	1.009	0.31
	SE	2.152	0.332	0.155	0.000	1.016	0.17
	BCVA	− 6.055	5.547	− 0.026	0.275	1.02	− 0.07
	Gender	− 0.558	0.842	− 0.016	0.508	1.004	− 0.02
GCIPL	Image quality	0.426	0.026	0.375	0.000	1.009	0.39
	SE	0.989	0.106	0.213	0.000	1.016	0.23
	BCVA	− 3.492	1.773	− 0.045	0.049	1.02	− 0.10
	Gender	0.367	0.269	0.031	0.173	1.004	0.05
	Height	0.041	0.022	0.042	0.066	1.002	0.05
GCC	Image quality	0.372	0.043	0.212	0.000	1.009	0.22
	SE	0.684	0.177	0.095	0.000	1.016	0.11
	BCVA	0.086	0.037	0.057	0.020	1.002	− 0.12
	Gender	− 10.557	2.948	− 0.088	0.000	1.02	0.05
	Height	0.637	0.448	0.035	0.155	1.004	0.06
RNFL	Image quality	− 0.114	0.031	− 0.094	0.000	1.009	− 0.09
	SE	− 0.290	0.125	− 0.059	0.021	1.016	− 0.05
	BCVA	− 7.116	2.090	− 0.087	0.001	1.02	− 0.07
	Gender	0.265	0.317	0.021	0.403	1.004	0.01

*P < 0.05, significantly correlated parameters.

Upon adjusting for age, sex, height, and weight, Pearson’s correlation analysis portrayed that the value of SE was positively correlated with the thickness of most retinal regions, excluding the central and inferior regions of the inner annulus. Moreover, all regions of choroidal thickness were significantly correlated with SE (P < 0.001, r = 0.2–0.3), with the most robust correlation in the inner and outer sector of nasal region (r = 0.264, 0.257, respectively), succeeded by the inferior, superior, and temporal regions. For the thickness of GCIPL and GCC, only the outer annulus was more correlated with SE (P < 0.01), but the inner annulus had no apparent correlation. RNFL thicknesses in the average, inner-inferior, and nasal regions were negatively related to SE (P < 0.05). (Table 3).

**Table 3:** Correlation between retinal, choroidal sub-region thickness and SE.

Region	Center	Inner_T	Inner_S	Inner_N	Inner_I	Outer_T	Outer_S	Outer_N	Outer_I	Average
Choroid										
r value	0.239	0.213	0.22	0.264	0.224	0.213	0.228	0.257	0.233	0.267
P value	< 0.001*	< 0.001*	< 0.001*	< 0.001*	< 0.001*	< 0.001*	< 0.001*	< 0.001*	< 0.001*	< 0.001*
Full retina										
r value	0.017	0.087	0.06	0.083	0.01	0.156	0.096	0.145	0.117	0.124
P value	0.497	0.001*	0.018*	0.001*	0.684	< 0.001*	< 0.001*	< 0.001*	< 0.001*	< 0.001*
Ganglion cell-inner plexiform layer										
r value	− 0.008	0.051	0.041	0.084	− 0.001	0.175	0.129	0.234	0.16	0.19
P value	0.752	0.042*	0.107	0.001*	0.964	< 0.001*	< 0.001*	< 0.001*	< 0.001*	< 0.001*
Ganglion cell complex										
r value	− 0.008	0.055	0.022	0.022	− 0.037	0.156	0.069	0.076	0.102	0.093
P value	0.744	0.029*	0.384	0.392	0.143	< 0.001*	0.007*	0.003*	< 0.001*	< 0.001*
Retinal nerve fiber layer										
r value	− 0.009	0.015	− 0.016	− 0.068	− 0.057	0.025	− 0.031	− 0.109	− 0.011	− 0.051
P value	0.717	0.547	0.524	0.007a	0.025a	0.333	0.224	< 0.001a	0.658	0.046a

*T* temporal, *S* superior, *N* nasal, *I* inferior.

*Thickness of regions significantly associated with spherical equivalent refraction in the Pearson correlation analysis.

### Progression and change in macular thickness after 20 months

The alterations in the retinal thickness of Lhasa children after nearly 2 years (20 months), compared to the baseline examination, are illustrated in Table 4. Excluding the inner-nasal, outer-inferior, and central regions, thickness of the full retina decreased significantly (P < 0.05). The average thickness of GCIPL also decreased, but GCC exhibited no significant change. Changes in retinal thickness between the two years were compared with refractive groups at baseline (Table 5). The myopic group demonstrated larger changes than the hyperopic group in regions of a thinning retina and fewer changes in regions of a thickening retina, but without a statistically significant difference. Correlation analysis depicted that the change in SE was positively correlated solely with the change in GCIPL thickness (r = 0.10, P = 0.002) but not with the thickness of other areas of the retina.

**Table 4:** Paired t-test for changes in retinal thickness between 2 years.

Macular layers		Mean	SD	95% CI		t	P value
Full retina (μm)	Center	− 32.54	31.10	− 34.58	− 30.51	− 31.35	< 0.001*
	Average	5.84	18.63	4.62	7.06	9.39	< 0.001*
	Inner_T	3.86	21.96	2.42	5.30	5.27	< 0.001*
	Inner_S	4.86	24.29	3.26	6.45	5.99	< 0.001*
	Inner_N	− 4.92	25.08	− 6.56	− 3.28	− 5.88	< 0.001*
	Inner_I	15.64	28.90	13.75	17.53	16.22	< 0.001*
	Outer_T	9.65	22.35	8.19	11.11	12.94	< 0.001*
	Outer_S	10.50	20.27	9.17	11.82	15.52	< 0.001*
	Outer_N	11.87	22.24	10.41	13.32	16.00	< 0.001*
	Outer_I	− 1.74	26.27	− 3.46	− 0.02	− 1.99	0.047*
GCIPL (μm)	Average	5.22	5.30	4.87	5.57	29.46	< 0.001*
GCC (μm)	Average	− 0.13	8.74	− 0.71	0.44	− 0.46	0.649

*P < 0.05, Statistical differences between the thickness in the baseline and second-year follow-up examination.

**Table 5:** Comparison of retinal thickness changes between different refractive errors (grouped by baseline examination).

Macular layers		Myopia (n = 32)		Emmetropia (n = 805)		Hyperopia (n = 61)		F value	P value
		Mean	SD	Mean	SD	Mean	SD		
Full retina (μm)	Center	31.73	22.41	32.41	31.65	34.78	27.91	0.18	0.84
	Inner_T	− 7.39	8.65	− 4.05	22.79	0.45	13.64	1.62	0.20
	Inner_S	− 6.16	9.02	− 5.25	25.35	1.06	11.39	1.97	0.14
	Inner_N	2.40	14.44	4.53	25.90	11.36	16.10	2.28	0.10
	Inner_I	− 14.66	18.43	− 15.93	29.88	− 12.31	18.29	0.46	0.63
	Outer_T	− 10.08	10.67	− 9.83	23.31	− 7.11	10.83	0.42	0.65
	Outer_S	− 10.62	15.57	− 10.81	21.05	− 6.25	7.72	1.44	0.24
	Outer_N	− 14.55	9.13	− 12.15	23.13	− 6.77	12.24	1.90	0.15
	Outer_I	0.49	9.23	1.62	27.37	3.99	15.07	0.27	0.77
	Average	− 5.84	5.22	− 6.07	19.54	− 2.78	7.17	0.89	0.41
GCIPL (μm)	Average	− 4.60	3.15	− 5.29	5.48	− 4.64	3.32	0.64	0.53
GCC (μm)	Average	0.27	3.78	0.08	9.08	0.74	5.25	0.16	0.85

## Discussion

To the best of our knowledge, this is the sole cohort study to broadly discuss the thickness of the choroid and various retina layers in the detailed macular sub-regions of children in Lhasa, Tibet. In the baseline examination of LCES, we meticulously described the macular retinal and circumpapillary RNFL thickness^8^. In the second year of follow-up, we measured and compared the ChT and retinal thickness of Lhasa children. The observations include the following: (1) The thickness of the choroid and retina was significantly reduced in myopic children. The myopic group demonstrated more pronounced changes than the hyperopic children in regions of retinal thinning and fewer changes in regions of retinal thickening. (2) The value of SE exhibited a positive correlation with all regions of choroidal and retinal thickness, excluding the central and inner inferior regions of the full retina. (3) Every choroidal subregion was significantly correlated with the thickness of the full retina, excluding the central region. (4) The thickness of the choroid, GCIPL, and GCC of girls were significantly thicker than those of boys. The average and central thickness of the full retina and choroid showed no significant correlation with age, height, weight, or BMI. (5) Compared to baseline data from 20 months earlier, most regions of the full retina had significantly decreased. The change in SE was positively associated solely with the change in GCIPL thickness.

In previous studies, Nagasawa et al.^24^ reported that the central ChT of eight-year-old children in Japan with the SE of − 0.04 ± 0.96D was approximately 260.4 ± 57.2 μm. Also, Peiyao Jin et al. discovered that the average ChT of students in Shanghai was 251 ± 62 mm (SE = 0.20D, AL = 23.18 mm); also in studies of children in Australia (ChT = 350 μm)^25^, Queensland (ChT = 337 ± 65 μm)^26^, Shandong in China (ChT = 283 ± 67 μm)^27^, ChT were thicker than that in the current study. It is generally accepted that the choroid progressively becomes thinner as myopia advances^2,28^. Despite higher values of SE and shorter AL, the ChT in this study remains thinner than those observed in Shandong^27^ and Inner Mongolia^29^ (SE =  − 1.41D, − 1.20D respectively) in China. We hypothesize that this difference may be attributed to ethnic variations. This research revealed that, after adjusting for AL, the choroidal thickness of the myopic group was significantly less than that of the hyperopic and emmetropic groups, with the difference being most noticeable in the central and nasal regions of both the inner and outer annulus. Jin et al.^4^ reported similar findings, but they proposed that choroidal thinning was more evident in myopic eyes in the superior and inferior perifoveal regions. We also found a significant positive correlation between choroidal thickness and the value of SE, with the most significant correlation in the nasal region. The choroidal thinning of myopic group was more pronounced in the nasal area where the choroid was originally thinner in distribution, indicating that the nasal area may be a more sensitive region for predicting the onset of early myopia. It appears that choroidal thinning occurs early in myopic progression, possibly before axial elongation, implying that ChT thinning may act as an earlier biomarker of myopia progression^30,26^.

While we did not observe significant changes in the choroidal perifoveal area in myopic eyes, we did identify such results in the thinning of the retina, particularly the GCC and GCIPL layers. Previous studies have produced inconsistent evidence on the relationship between retinal thickness and myopia. Some studies^31,32^ have reported that the retinal thickness of myopic patients is significantly less. Some found that the retinal thickness is centrally thickened and peripherally thinned^28,33^. In this study, the macular retinal thickness of myopic patients was less than that of emmetropic and hyperopic patients in all sub-regions except the central region. Moreover, the average retinal thickness also demonstrated a significant positive correlation with SE, which is consistent with the conclusions of our baseline study^8^. The mean and outer-sector thickness of GCC and GCIPL in this study were thinner in myopic students, and both were positively correlated with SE. Jin et al.^34^ and Yoo et al.^35^ also found that outer-sector GCC thickness was positively correlated with SE, whereas inner-sector was not. This implies that the thickness of the outer-sector GCC and GCIPL is more sensitive to SE changes and may be considered potential risk factors for myopia progression, requiring long-term monitoring and follow-up. The difference in thickness of the RNFL layer was insignificant between refractive groups, but SE was negatively correlated with the nasal and mean thickness of RNFL in this study.

Serving as the source of retinal blood supply and oxygen provision, choroidal thickness is also significantly correlated with retinal thickness. This study discovered that the thickness of each choroidal region was significantly correlated with retinal thickness, excluding the central region. The correlation is slightly higher at the outer annulus. Jin et al.^4^ reported that retinal thinning is observed later during the progression of myopia. Considering the systemic factors influencing the choroid, changes in retinal thickness may be estimated more reliably. This suggests that changes in retinal thickness may also serve as a monitoring factor for the onset or progression of myopia^2^. However, changes in retinal thickness may occur later than changes in choroidal blood flow and thickness; thus, it may be designated as a follow-up and prognostic indicator for myopia, despite its low sensitivity.

A clear correlation between choroidal thickness and oxygen saturation was not found in this study, but a positive correlation was found with MABP. The choroid, as a vascular layer in the eye, is susceptible to blood flow-related factors such as blood pressure^36,37^. Zhu et al.^29^ also reported a negative correlation between subfoveal choroidal thickness and SBP and DBP in children. Thus the association between choroidal thickness and blood pressure may require further follow-up and demonstration. ChT was not significantly associated with systemic factors such as age, height, and weight in our study. While retinal thickness of different layers was positively correlated with weight. Additionally, we discovered that stereoacuity was negatively associated with GCIPL and GCC thickness. Stereoacuity gradually develops to the adult level in childhood, which was correlated to binocular vision and amblyopia^38,39^. The strong correlation between BCVA and stereoacuity was also found in the present study. Therefore, in the multivariate analysis we included only BCVA and excluded stereoacuity. Thinning of GCIPL and GCC is generally associated with glaucoma progression^40–42^, but its relevence to amblyopia is inconclusive^43–45^. Thinning of GCC and GCIPL may induce stereopsis disorder due to affecting visual field or potential visual function, but this deduction requires further research for confirmation.

Comparing the changes in retinal thickness between the baseline and the second year of follow-up, it was found that the retinal thickness of Lhasa children was significantly reduced, except for the central area, which had thickened. GCC and GCIPL were also significantly thinner. Correlation analysis demonstrated that the change in GCIPL was significantly positively correlated with the change in SE, but no clear correlation was found between full retinal and GCC thickness. Similar to the above conclusions, Jin^34^ reported that the 1-year change in SE was correlated only with outer retinal layer (ORL) change but not with full retina and GCC. Although retinal thickness reduces with decreasing SE value, this change does not exhibit a clear dose–response relationship.

Despite the undeniable advantages of this study—for instance, a large-scale sample, minority subjects, standardized examination, integrated OCT data, and intensive analysis—it also has some limitations. Due to the lack of local medical equipment, we used a different machine to measure AL at baseline. Equipment changes may have affected our data comparisons at follow-up. Therefore, statistical analysis of baseline AL data was not included in this study. Due to the small sample proportion of children of Han and other ethnic groups, differences in the thickness and development of the retina and choroid between ethnic groups are not statistically comparable. Children’s collaboration with OCT testing has not been greatly enhanced due to language barrier. These problems may be resolved by increasing the sample in further follow-up.

In conclusion, this study illustrates that SS-OCT can effectively obtain extensive ophthalmic biometry data on Tibetan children. The choroidal thickness of Tibetan children is thinner than that of same-age children from other regions. A thicker choroid was associated with higher values of SE, best-corrected vision acuity, and mean arterial blood pressure in multivariate linear regression. Choroid thinning may serve as a preliminary biomarker of myopia progression. Thinning of the retina, the outer-sector GCC and GCIPL may be designated as a follow-up and prognostic indicator for myopia, despite their lower sensitivity. The findings may aid in establishing a standardized Tibetan children’s ophthalmology database, particularly to illustrate the relationship between the retinal thickness of different layers and refractive status. Our current study further suggests that routine OCT screening may be effective for the long-term monitoring of ocular diseases in the plateau of China.

## Methods

### Research subjects and design

The Lhasa Childhood Eye Study (LCES) is an epidemiological cohort study that included grade-1 students from seven elementary schools in Lhasa, Tibet, over a 5-year follow-up period until they entered junior high school. At baseline in 2019, 1856 Tibetan students, with an average age of 6.83 years, were included in the cohort and examined yearly. The research protocol complied with the Declaration of Helsinki and was approved by the Ethics Committee of Beijing Tongren Hospital, Capital Medical University (No. TRECKY2019-058). An exhaustive ophthalmic examination was performed according to the standard operating procedures and supervised by an ophthalmologist and an epidemiologist. Before the examination, parents or guardians of all participating children had signed informed consent forms. The study design and protocols for LCES have been published elsewhere^46^. The data of this study were obtained from the baseline and the second-year follow-up in 2019 and 2021, respectively.

### Ocular examinations and definitions

All participants were subjected to a comprehensive ophthalmic examination of both eyes, encompassing the measurement of uncorrected visual acuity and best-corrected visual acuity (BCVA) (250300; Goodlite, Elgin, IL, USA), stereoacuity (S0001, STEREO, USA), ocular dominance, slit-lamp facilitated biomicroscopy (SL-3G, Topcon, Tokyo, Japan), tonometry (CT-800, Topcon, Tokyo, Japan), ocular motility, auto refractometer prior to and following cycloplegia, axial length (AL) (IOL master, ZEISS, Germany in 2021, Lenstar, Haag-Streit, Switzerland in 2019), OCT, and retinal photographs. Moreover, all students underwent measurements for height, weight, blood pressure, heart rate, and blood oxygen saturation. Before applying the cycloplegia medication, slit-lamp examination was performed to ensure that there was no risk of mydriasis. Then each participant without contraindication was first administered one drop of topical anesthetic agent (Alcaine, Alcon) to alleviate discomfort, followed by two drops of 1% cyclopentolate (Alcon) and 1 drop of Mydrin P (Santen) after a 5-min interval. Thirty minutes after the last drop, a third drop of 1% cyclopentolate was administered whenever pupillary light reflex was still present or where the pupil size was less than 6.0 mm, and the examination was repeated 15 min later. If complete cycloplegia was not achieved after the third drop, subjects did not undergo cycloplegic refraction.

The data for AL, intraocular pressure (IOP), and optometry were averaged over three measurements. Spherical equivalent (SE) was computed as spherical power plus half of the cylindrical power, which was derived from cycloplegic optometry. Myopia, emmetropia, and hyperopia were classified as SE ≤  − 0.50 diopters (D), − 0.50D < SE < 0.50D, and SE ≥ 0.5D respectively in at least one eye. Stereoacuity was categorized as normal (= 40 arcsec), equivocal (> 40 and ≤ 100 arcsec), subnormal (> 100 and ≤ 400 arcsec), and abnormal stereoacuity (> 400 arcsec)^47^. The body mass index (BMI) was calculated using the ratio of body weight (kg) to the square of body height (m). Mean arterial blood pressure (MABP) was computed as two-thirds of the diastolic blood pressure (DBP) plus one-third of the systolic blood pressure (SBP).

### OCT measurements

**Figure 2:**
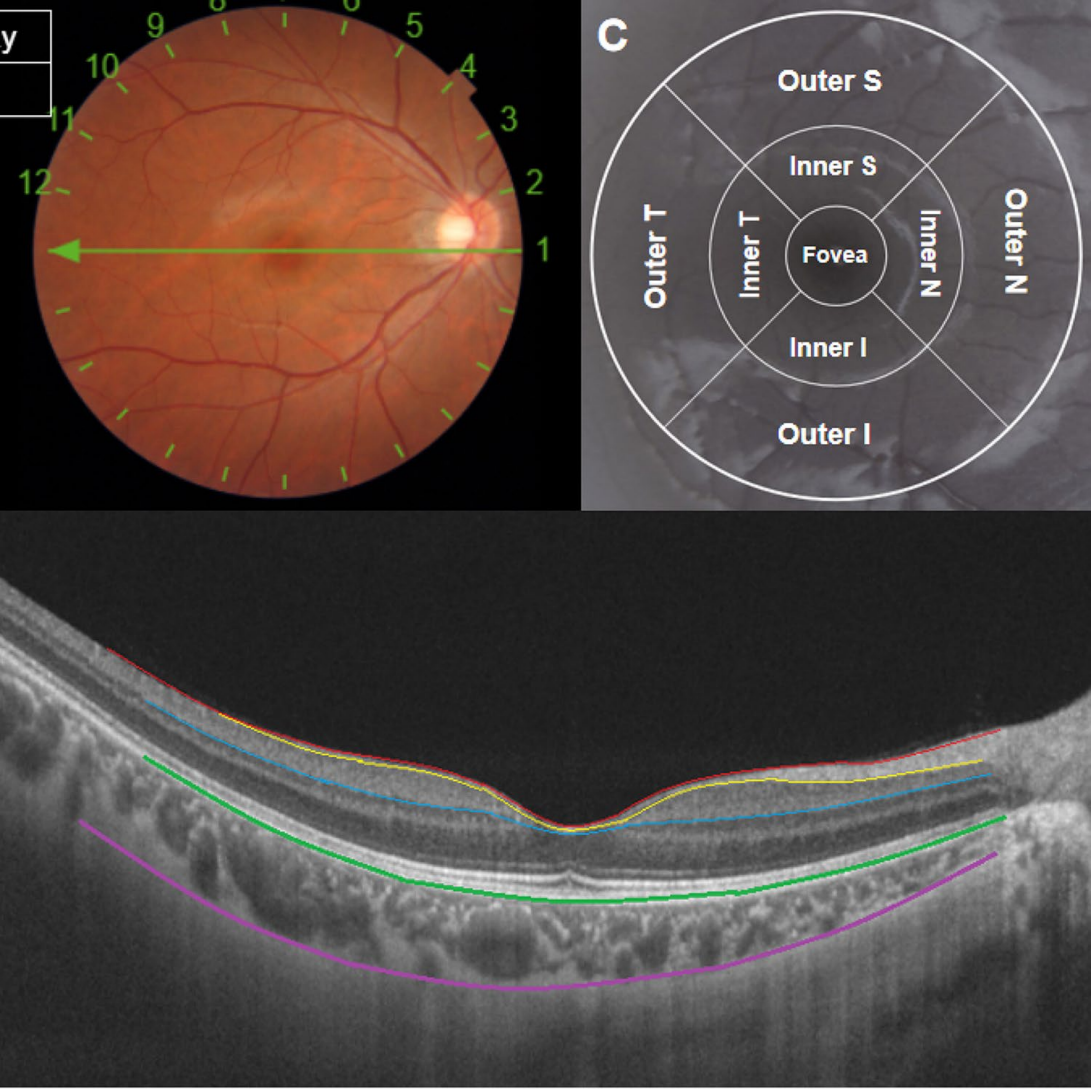
The Radial 9 × 9 mm scan region (**A**) and macula ETDRS grid, (**B**) overlaid with example projected images. (**C**) Full retinal thickness (distance between the red and green boundaries); ganglion cell + inner plexiform layers (distance between the yellow and blue boundaries); ganglion cell complex (distance between the red and blue boundaries, vitreal to inner nuclear layer); retinal nerve fiber layer (distance between the red and yellow boundaries), choroid (distance between the green and purple boundaries).

A experienced technician employed SS-OCT (DRI OCT Triton-1, Topcon, Japan) to gauge the thickness of the retina and choroid layers. Given that choroidal thickness is influenced by circadian rhythms and cycloplegia, all children were examined prior to cycloplegia, between 1 p.m. and 4 p.m. In this study, we opted for the ‘Radial 9 × 9 scan mode’, where the macula was designated as the center point with a 9 mm diameter and a 12-line radial scan patterns with 100,000 A-scans per second. The macula was partitioned, in accordance with the ETDRS map, into three annuli with diameters of 1 mm (center), 3 mm (inner), and 6 mm (outer). The inner and outer annuli were further divided into four quadrants: temporal, superior, nasal, and inferior. The segment and thickness of each layer of the retina and choroid were automatically evaluated by the SS-OCT device. Selected for analysis in this study were the thickness of the full retina, retinal nerve fiber layer (RNFL, the inner limiting membrane to the interface between the nerve fiber layer and ganglion cell layer), GCIPL (the interface between the RNFL and ganglion cell layer to the interface between the inner plexiform layer and inner nuclear layer), GCC (the aggregate of RNFL and GCIPL), and choroid (Bruch membrane to the choroid-sclera interface) (Fig. 2).

For this study, because the choroid regions and retinal layers were automatically segmented by the instrument, only images of sufficient quality were included in the following evaluation. Therefore, data were excluded from image acquisition by manual examination if the images were substandard (signal strength below 60, blinking or motion artifacts, wrong automatic segmentation, etc.)^48^. All our examinations in this study were performed in both eyes. Considering that the binocular data consistency was high (r = 0.95), we selected only the right healthy eye data for analysis.

### Statistical analysis

Data were presented as mean ± standard deviation for continuous variables and as median (quartile) for categorical variables. The IBM SPSS Statistics analysis software (version 24.0) was utilized. An independent samples t-test and variance analysis were employed to contrast differences among retinal and choroidal thickness concerning genders and refractive groups. Covariance analysis was applied to eliminate the effect of confounding factors such as AL and IOP. Univariate and multivariate linear regression analysis was used to estimate the effects of sex, age, BMI, heart rate, blood oxygen saturation, MABP, uncorrected VA, BCVA, near VA, IOP, stereoacuity, and SE on retinal and choroidal thickness. Pearson’s correlation analysis was utilized to evaluate the association between retinal and choroidal thickness. A paired t-test was implemented to compare the changes in macular retinal thickness between the baseline and follow-up. Repeated-measures ANOVA was used to contrast the differences in retinal thickness changes between refractive groups. A two-tailed P value of less than 0.05 was defined as statistically significant at the 95% confidence interval level.

### Statement of ethics

The study protocols adhered to the declaration of Helsinki and were approved by the Ethics Committee of the Beijing Tongren Hospital, Capital Medical University (No. TRECKY2019-058). All participants’ parents or legal guardians gave written informed consent prior to the study.

### Supplementary Information

Supplementary Table 1.

## Data Availability

All data generated or analyzed during this study are included in this article and its Supplementary Material files. Further enquiries can be directed to the corresponding author.
