# Supplementary material for: Assessment of macular choroidal and retinal thickness: a cohort study in Tibetan healthy children
Source: Sci Rep. 2024 Jan 16;14:1383. doi: 10.1038/s41598-024-51949-0 (PMC10792070; doi:10.1038/s41598-024-51949-0)
Supplement: Supplementary file 1 — Supplementary Table 1. [file 41598_2024_51949_MOESM1_ESM.doc]

**Table 1** **Sex-specific distribution and refraction group of macular choroid and retinal thickness**

| **Thickness of macular layers, μm** | | **Boys(n=812)** | | **Girls(n=743)** | | **T value** | **P value** | **Myopia(n=337)** | | **Emmetropia(n=1180)** | | **Hyperopia(n=38)** | | **F value** | **P value** |
| --- | --- | --- | --- | --- | --- | --- | --- | --- | --- | --- | --- | --- | --- | --- | --- |
|  |  | **Mean** | **SD** | **Mean** | **SD** |  |  | **Mean** | **SD** | **Mean** | **SD** | **Mean** | **SD** |  |  |
| **Choroid** | Center | 244.07 | 53.22 | 253.55 | 56.72 | -3.41 | 0.001^*^ | 225.01 | 52.14 | 254.19 | 53.65 | 285.90 | 60.04 | 49.11 | 0.001^*^ |
|  | Inner_T | 248.41 | 54.65 | 257.44 | 56.82 | -3.21 | 0.001^*^ | 229.32 | 49.75 | 258.82 | 55.29 | 272.52 | 67.82 | 41.62 | 0.001^*^ |
|  | Inner_S | 239.00 | 53.91 | 246.86 | 55.18 | -2.85 | 0.004^*^ | 221.60 | 51.84 | 247.65 | 53.36 | 280.13 | 63.12 | 41.39 | 0.004^*^ |
|  | Inner_N | 221.29 | 50.74 | 229.84 | 53.46 | -3.25 | 0.001^*^ | 202.36 | 46.58 | 230.47 | 51.41 | 272.88 | 51.12 | 58.74 | 0.001^*^ |
|  | Inner_I | 249.24 | 56.18 | 257.50 | 58.04 | -2.87 | 0.004^*^ | 231.18 | 52.55 | 258.55 | 56.42 | 283.54 | 68.25 | 37.63 | 0.004^*^ |
|  | Outer_T | 246.74 | 51.77 | 254.89 | 52.99 | -3.08 | 0.002^*^ | 229.13 | 46.18 | 256.28 | 52.04 | 267.38 | 67.99 | 39.42 | 0.002^*^ |
|  | Outer_S | 235.35 | 50.02 | 241.35 | 49.58 | -2.39 | 0.017^*^ | 219.53 | 47.08 | 242.36 | 48.90 | 276.58 | 54.49 | 41.43 | 0.017^*^ |
|  | Outer_N | 183.17 | 43.67 | 189.10 | 45.46 | -2.63 | 0.009^*^ | 169.19 | 37.70 | 189.43 | 44.34 | 230.23 | 56.16 | 49.39 | 0.009^*^ |
|  | Outer_I | 240.47 | 51.43 | 244.85 | 51.11 | -1.69 | 0.092 | 222.89 | 46.66 | 247.40 | 50.47 | 268.55 | 68.31 | 36.99 | 0.092 |
|  | Average | 229.74 | 43.46 | 236.45 | 44.85 | -3.01 | 0.003^*^ | 212.94 | 38.86 | 237.67 | 43.43 | 265.03 | 54.65 | 55.45 | 0.003^*^ |
| **GCIPL** | Center | 52.67 | 15.80 | 52.36 | 16.80 | 0.38 | 0.706 | 52.90 | 15.10 | 52.43 | 16.59 | 51.95 | 16.86 | 0.13 | 0.706 |
|  | Inner_T | 83.41 | 10.79 | 82.38 | 10.46 | 1.91 | 0.056 | 82.04 | 11.07 | 83.09 | 10.59 | 85.68 | 7.27 | 2.61 | 0.056 |
|  | Inner_S | 87.66 | 12.52 | 87.34 | 10.47 | 0.56 | 0.579 | 86.46 | 12.22 | 87.76 | 11.45 | 89.08 | 9.54 | 2.04 | 0.579 |
|  | Inner_N | 90.28 | 10.69 | 89.59 | 10.07 | 1.32 | 0.188 | 88.32 | 11.80 | 90.39 | 10.02 | 90.93 | 6.72 | 5.50 | 0.188 |
|  | Inner_I | 82.09 | 15.02 | 81.49 | 12.99 | 0.85 | 0.394 | 81.61 | 14.28 | 81.79 | 14.17 | 83.98 | 8.80 | 0.49 | 0.394 |
|  | Outer_T | 69.73 | 9.46 | 70.18 | 8.94 | -0.96 | 0.338 | 67.75 | 9.60 | 70.43 | 9.05 | 74.66 | 6.57 | 16.70 | 0.338 |
|  | Outer_S | 61.45 | 9.40 | 62.32 | 8.77 | -1.88 | 0.061 | 59.95 | 8.93 | 62.27 | 9.10 | 66.35 | 8.29 | 13.61 | 0.061 |
|  | Outer_N | 69.07 | 8.75 | 70.28 | 8.75 | -2.74 | 0.006^*^ | 66.40 | 8.99 | 70.38 | 8.46 | 76.05 | 7.39 | 39.68 | 0.006^*^ |
|  | Outer_I | 65.63 | 12.12 | 68.36 | 10.72 | -4.71 | <0.001^*^ | 64.38 | 11.57 | 67.48 | 11.50 | 72.65 | 8.54 | 14.63 | <0.001^*^ |
|  | Average | 70.40 | 7.40 | 71.23 | 6.75 | -2.31 | 0.021^*^ | 68.73 | 7.58 | 71.25 | 6.88 | 75.19 | 4.89 | 24.85 | 0.021^*^ |
|  |  |  |  |  |  |  |  |  |  |  |  |  |  |  |  |
| **GCC** | Center | 62.54 | 21.88 | 62.36 | 23.40 | 0.16 | 0.872 | 62.86 | 21.38 | 62.37 | 22.94 | 61.43 | 23.64 | 0.10 | 0.872 |
|  | Inner_T | 106.20 | 13.32 | 104.79 | 13.16 | 2.10 | 0.036^*^ | 103.73 | 13.83 | 106.02 | 13.20 | 106.30 | 7.55 | 4.03 | 0.036^*^ |
|  | Inner_S | 118.90 | 17.11 | 118.88 | 14.26 | 0.02 | 0.982 | 117.75 | 16.64 | 119.16 | 15.69 | 120.79 | 10.98 | 1.34 | 0.982 |
|  | Inner_N | 117.02 | 14.60 | 116.49 | 13.50 | 0.74 | 0.457 | 115.96 | 16.30 | 117.07 | 13.52 | 114.97 | 8.73 | 1.14 | 0.457 |
|  | Inner_I | 108.47 | 22.43 | 106.49 | 19.25 | 1.87 | 0.062 | 108.10 | 21.54 | 107.42 | 21.05 | 105.82 | 12.90 | 0.27 | 0.062 |
|  | Outer_T | 94.71 | 11.80 | 95.23 | 11.99 | -0.87 | 0.385 | 91.82 | 12.84 | 95.77 | 11.58 | 97.71 | 6.83 | 16.04 | 0.385 |
|  | Outer_S | 104.99 | 14.45 | 107.85 | 12.11 | -4.22 | <0.001^*^ | 104.55 | 14.31 | 106.71 | 13.30 | 111.51 | 7.47 | 6.33 | <0.001^*^ |
|  | Outer_N | 120.85 | 14.03 | 122.29 | 12.43 | -2.14 | 0.033^*^ | 119.24 | 15.71 | 122.13 | 12.61 | 123.70 | 8.08 | 6.84 | 0.033^*^ |
|  | Outer_I | 108.64 | 18.85 | 111.66 | 14.21 | -3.56 | <0.001^*^ | 106.94 | 18.00 | 110.91 | 16.59 | 112.26 | 10.92 | 7.77 | <0.001^*^ |
|  | Average | 107.28 | 12.31 | 108.53 | 10.17 | -2.18 | 0.03^*^ | 105.77 | 13.08 | 108.42 | 10.86 | 110.10 | 6.42 | 8.05 | 0.03^*^ |
| **Full retina** | Center | 233.08 | 35.17 | 232.98 | 33.66 | 0.06 | 0.953 | 232.07 | 34.68 | 233.21 | 34.53 | 236.42 | 29.95 | 0.33 | 0.953 |
|  | Inner_T | 286.13 | 28.60 | 284.12 | 24.02 | 1.50 | 0.134 | 280.75 | 31.63 | 286.21 | 24.99 | 292.48 | 16.95 | 7.17 | 0.134 |
|  | Inner_S | 299.13 | 36.29 | 299.06 | 28.17 | 0.04 | 0.967 | 295.36 | 34.50 | 299.90 | 32.42 | 307.57 | 16.80 | 3.90 | 0.967 |
|  | Inner_N | 300.67 | 29.79 | 299.16 | 24.02 | 1.10 | 0.272 | 295.70 | 34.65 | 301.01 | 24.85 | 305.40 | 14.55 | 5.88 | 0.272 |
|  | Inner_I | 286.24 | 39.92 | 284.07 | 29.96 | 1.21 | 0.226 | 283.88 | 37.45 | 285.43 | 35.37 | 290.41 | 19.01 | 0.67 | 0.226 |
|  | Outer_T | 255.77 | 27.31 | 255.54 | 22.53 | 0.18 | 0.855 | 249.18 | 30.99 | 257.25 | 23.11 | 264.50 | 15.15 | 16.45 | 0.855 |
|  | Outer_S | 264.25 | 31.51 | 267.54 | 24.48 | -2.29 | 0.022^*^ | 261.12 | 29.73 | 266.78 | 28.21 | 277.99 | 12.26 | 8.93 | 0.022^*^ |
|  | Outer_N | 282.73 | 27.36 | 284.21 | 21.60 | -1.18 | 0.238 | 276.48 | 31.23 | 285.08 | 22.46 | 294.52 | 13.96 | 20.40 | 0.238 |
|  | Outer_I | 265.27 | 38.23 | 269.23 | 27.00 | -2.35 | 0.019^*^ | 260.40 | 38.06 | 268.80 | 32.04 | 276.72 | 19.49 | 10.11 | 0.019^*^ |
|  | Average | 271.89 | 27.72 | 273.16 | 20.55 | -1.02 | 0.307 | 267.04 | 29.32 | 273.77 | 23.06 | 281.87 | 12.62 | 13.01 | 0.307 |
| **RNFL** | Center | 10.02 | 7.53 | 10.08 | 7.69 | -0.16 | 0.872 | 10.16 | 7.74 | 10.04 | 7.58 | 9.43 | 7.36 | 0.16 | 0.872 |
|  | Inner_T | 23.13 | 9.23 | 22.57 | 10.05 | 1.15 | 0.252 | 22.11 | 7.22 | 23.15 | 10.33 | 20.60 | 3.14 | 2.63 | 0.252 |
|  | Inner_S | 31.64 | 9.55 | 31.78 | 8.63 | -0.31 | 0.756 | 31.79 | 9.67 | 31.69 | 8.95 | 31.69 | 9.65 | 0.02 | 0.756 |
|  | Inner_N | 27.14 | 8.18 | 27.17 | 8.99 | -0.07 | 0.948 | 28.14 | 10.43 | 26.97 | 8.03 | 24.01 | 5.19 | 5.08 | 0.948 |
|  | Inner_I | 26.82 | 14.61 | 25.19 | 11.03 | 2.48 | 0.013^*^ | 26.86 | 13.80 | 25.93 | 12.96 | 22.24 | 6.60 | 2.33 | 0.013^*^ |
|  | Outer_T | 25.25 | 7.80 | 25.19 | 9.54 | 0.13 | 0.897 | 24.46 | 6.81 | 25.51 | 9.24 | 23.04 | 2.25 | 3.18 | 0.897 |
|  | Outer_S | 44.00 | 8.84 | 45.75 | 7.57 | -4.19 | <0.001^*^ | 45.09 | 9.37 | 44.75 | 7.94 | 45.14 | 9.34 | 0.25 | <0.001^*^ |
|  | Outer_N | 52.42 | 10.27 | 52.29 | 10.09 | 0.26 | 0.796 | 53.85 | 9.79 | 52.08 | 10.29 | 47.69 | 8.11 | 8.14 | 0.796 |
|  | Outer_I | 43.63 | 12.79 | 43.49 | 10.49 | 0.24 | 0.808 | 43.34 | 10.66 | 43.74 | 12.13 | 40.07 | 7.47 | 1.88 | 0.808 |
|  | Average | 37.36 | 6.59 | 37.52 | 5.94 | -0.49 | 0.625 | 37.65 | 6.60 | 37.45 | 6.24 | 35.02 | 4.08 | 3.01 | 0.625 |

**Notes:** ^*^P<0.05, Statistical differences among the sex and refractive groups.
